# Paraneoplastic Encephalitis Unmasking Small-Cell Lung Cancer With Impending Superior Vena Cava Obstruction: A Case Report

**DOI:** 10.7759/cureus.100564

**Published:** 2026-01-01

**Authors:** Halima Zaman, Raksaini Sivasubramaniam, Zainab Akbar

**Affiliations:** 1 Oncology, Royal Preston Hospital, Preston, GBR; 2 Emergency, Royal Preston Hospital, Preston, GBR

**Keywords:** autoantibodies, autoimmune encephalitis, first episode psychosis, new-onset seizure, paraneoplastic encephalitis syndromes, small-cell lung carcinoma, superior vena cava (svc) obstruction

## Abstract

Small-cell lung cancer (SCLC) is an aggressive neuroendocrine carcinoma that accounts for approximately 15% of all lung cancers and is strongly associated with paraneoplastic syndromes. Among these, paraneoplastic encephalitis (PE) is a rare, immune-mediated inflammation that leads to neuropsychiatric symptoms, seizures, and memory impairment. It may precede the diagnosis of malignancy by weeks to months. Superior vena cava obstruction (SVCO), in contrast, is a more common oncological emergency. Their concurrent presentation as the initial manifestations of SCLC is an uncommon occurrence.

We report the case of a 57-year-old woman with a strong history of smoking, who presented with acute behavioral changes and seizures. Despite negative onconeural antibody testing, her presentation was strongly suggestive of autoimmune encephalitis. Immunotherapy with corticosteroids, plasma exchange, and intravenous immunoglobulin was trialed, but her neurological symptoms persisted. Subsequently, she developed neck swelling and dyspnea - imaging revealed mediastinal lymphadenopathy with venous compression concerning for impending SVCO. Endobronchial ultrasound-guided biopsy confirmed small-cell lung carcinoma. She was commenced on carboplatin-etoposide chemotherapy, which led to a clinical improvement in both neurological and obstructive symptoms.

This case highlights the diagnostic complexity of paraneoplastic encephalitis, which can mimic psychiatric or infectious causes. Awareness of this rare presentation is crucial, as timely identification and prompt oncological intervention can significantly improve outcomes in patients presenting with unexplained neuropsychiatric symptoms or new-onset seizures.

## Introduction

Small-cell lung cancer (SCLC) is an aggressive form of neuroendocrine carcinoma that accounts for approximately 15% of all lung cancers, with a strong association to cigarette smoking [1]. It is a rapidly growing tumor with early dissemination and poor prognosis. It has a five-year survival rate of less than 10% despite initial responsiveness to chemotherapy and radiotherapy [2]. Beyond its oncological burden, SCLC is notable for its systemic manifestations, particularly paraneoplastic syndromes and superior vena cava obstruction (SVCO) [3,4].

Paraneoplastic neurological syndromes (PNS) occur in up to 20% of patients with SCLC and result from immune cross-reactivity between the tumor antigens and neuronal tissue [3]. While paraneoplastic encephalitis is a rare manifestation, it is clinically significant - often presenting as seizures, neuropsychiatric symptoms, or cognitive impairment [5,6]. Among its forms, paraneoplastic limbic encephalitis (PLE) is most commonly linked to SCLC and frequently lacks detectable anti-Hu antibodies in up to 50% of cases, underscoring the diagnostic challenge in seronegative presentations [5]. Encephalitis may precede the diagnosis of the underlying tumor, creating another diagnostic challenge [6-8].

In contrast, SVCO is a relatively common oncological emergency in SCLC, occurring in approximately 8%-10% of cases [2]. It is the compression or invasion of the superior vena cava by bulky mediastinal disease and typically manifests with facial swelling, venous distension, and dyspnea [2]. Prompt recognition is crucial given its potential for life-threatening cardiorespiratory compromise [9].

The coexistence of paraneoplastic encephalitis and SVCO as presenting features of SCLC is distinctly uncommon. We report a unique case in which paraneoplastic encephalitis unmasked an underlying SCLC with an impending risk of SVCO. This case highlights the need for heightened clinical suspicion for occult malignancy in patients presenting with unexplained neurological syndromes, while also emphasizing vigilance for concurrent oncological emergencies.

## Case presentation

**Figure 1:**
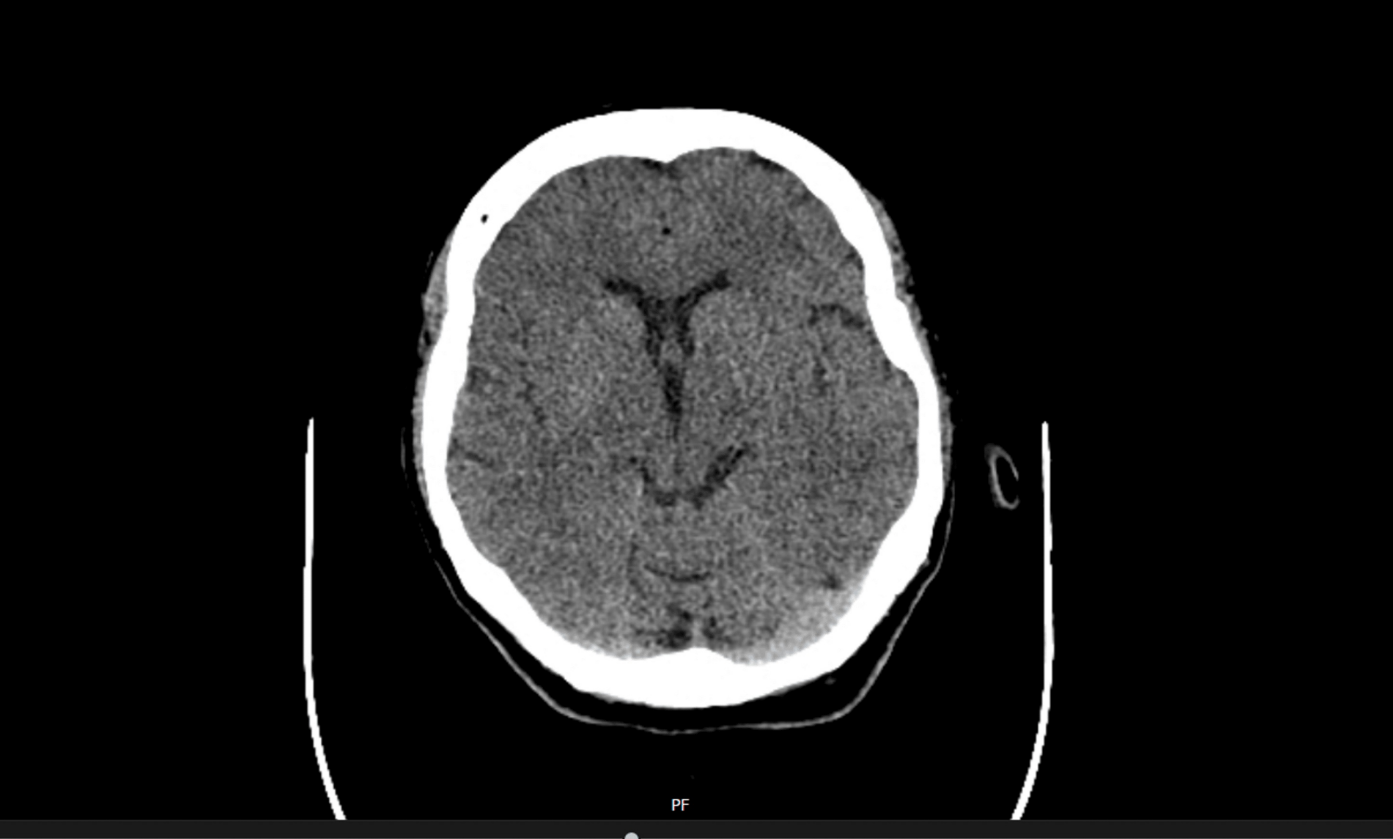
CT head demonstrating normal brain parenchyma CT: Computed tomography.

**Figure 2:**
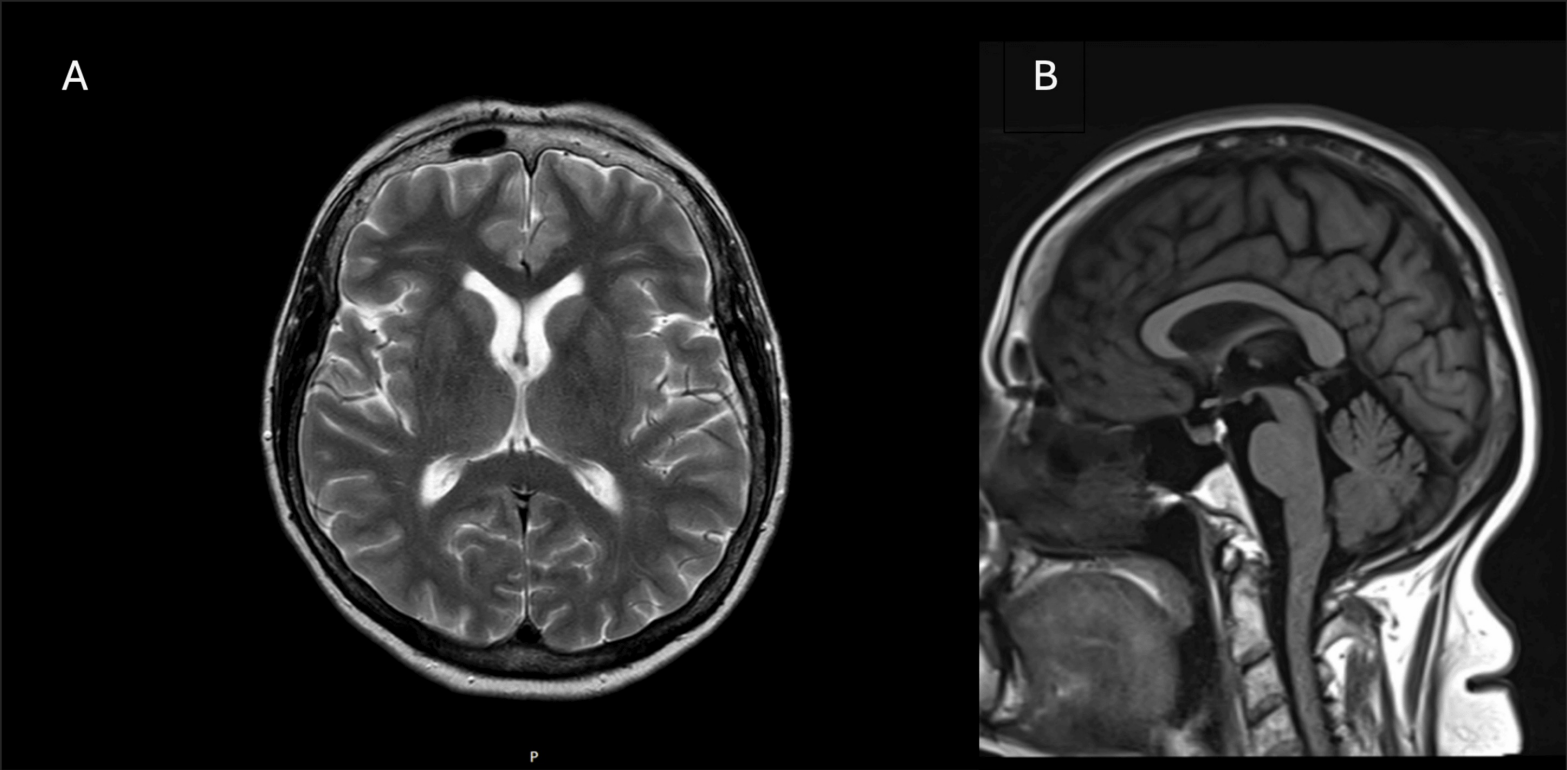
MRI brain demonstrating no abnormalities (A) Axial T2-weighted view and (B) T1-weighted sagittal view. MRI: Magnetic resonance imaging.

A 57-year-old lady presented to the emergency department with a four-day history of atypical behavior characterized by excessive spending, delusional thinking, hypomanic episodes, and periods of confusion. Her family reported that she had been exhibiting uncharacteristic behavior and making impulsive decisions. She has a background of depression, irritable bowel syndrome, and psoriasis, and has been actively smoking 20 cigarettes a day since the age of 20 and drinking 20-30 units of alcohol a week. She has no relevant family history, no history of trauma, and has worked as a carer. Bloods displayed mildly elevated inflammatory markers with a C-reactive protein (CRP) of 15.7 mg/L (normal range: 0-5 mg/L), white blood cells of 12.65 x 10^9^/L (normal range: 4-11 x 10^9^/L), and neutrophils of 8.10 x 10^9^/L (normal range: 1.6-7.5 x 10^9^) (Table 1). Both physical and neurological examinations were normal. Urine culture was positive for *Escherichia coli* (*E. coli*), and she was treated with a three-day course of antibiotics (nitrofurantoin). She was also started on Pabrinex vitamins B and C for her alcohol excess, and urine toxicology was negative. A CT head and magnetic resonance imaging (MRI) of the brain concluded that no significant abnormalities were seen (Figures 1, 2). She was admitted to a mental health facility under section 2 of the Mental Health Act (detention of an individual for up to 28 days for the purpose of assessment) and was treated with antipsychotics, benzodiazepines, and antidepressants for bipolar affective disorder - manic with psychosis (clonazepam, fluoxetine, and olanzapine).

**Figure 3:**
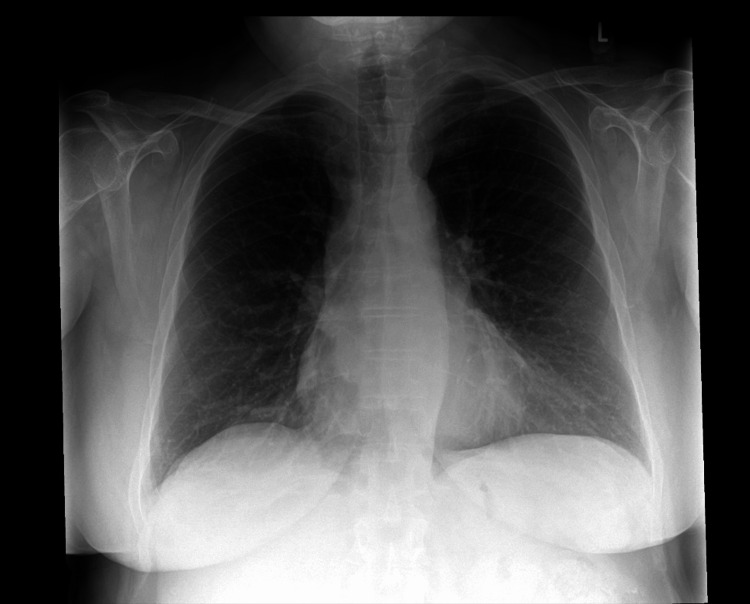
CXR demonstrating no acute lung or pleural abnormality CXR: Chest x-ray.

She re-presented to the emergency department seven weeks later from the psychiatric unit, detained under Section 3 of the Mental Health Act (for treatment of a mental disorder), following two generalized seizures. Both physical and neurological examinations were unremarkable, and she remained on the same psychotropic medications prescribed at discharge. Both blood tests (Table 1) and chest x-ray were unremarkable (Figure 3).

**Table 1:** Laboratory results CRP: C-reactive protein; WCC: White cell count.

Laboratory test	1st admission	2nd admission	Reference range
CRP	15.7	3.6	0-5 mg/L
WCC	12.65	11.11	4.0-11 x 10^9^ /L
Neutrophils	8.1	6.66	1.6-7.5 x 10^9^ /L
Hemoglobin	142	133	115-165 g/L
Platelets	318	361	140-440 x 10^9^ /L
Creatinine	49	44	45-84 μmol/L

**Figure 4:**
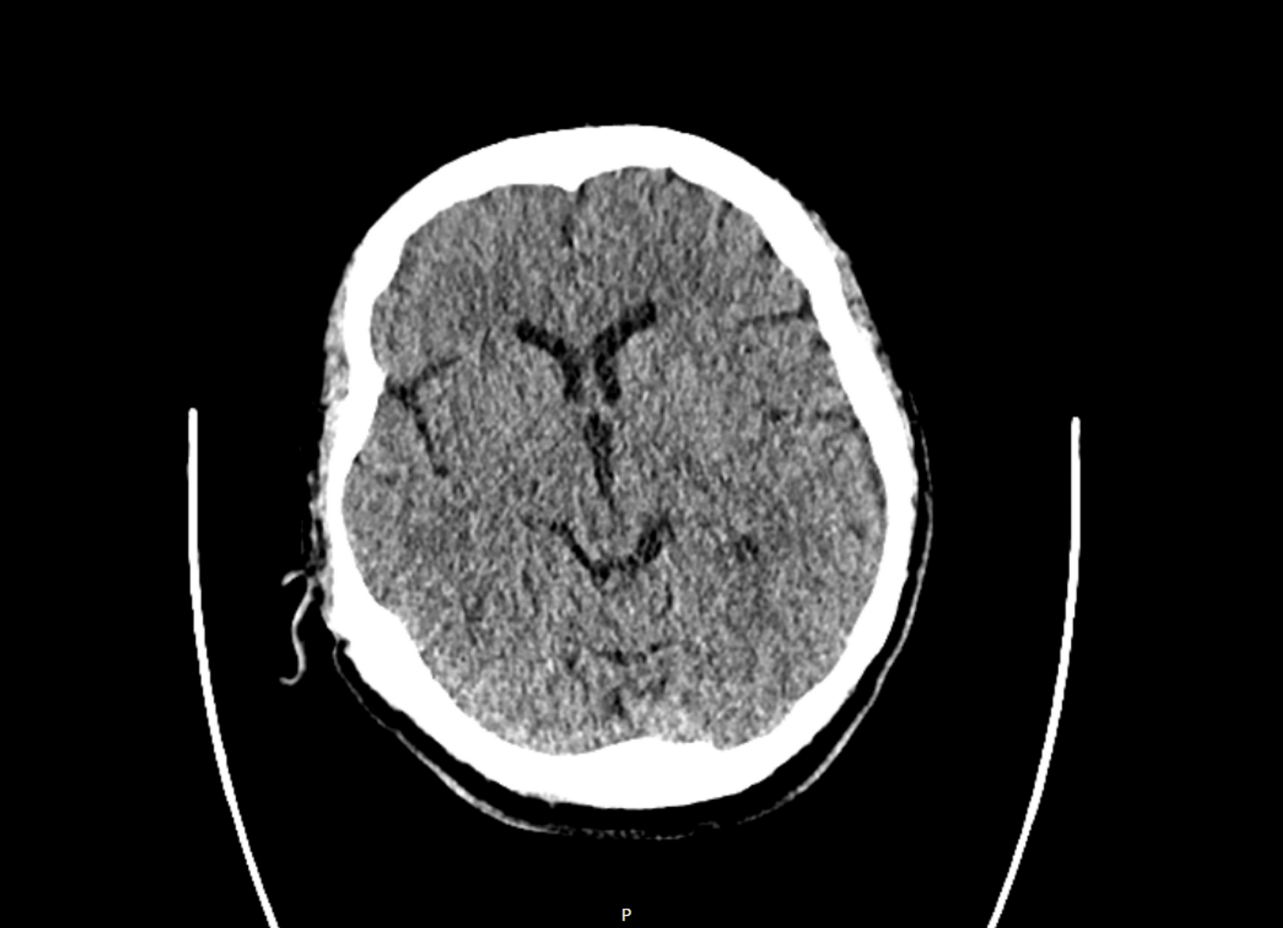
Repeat CT head demonstrating no intracranial abnormalities

**Figure 5:**
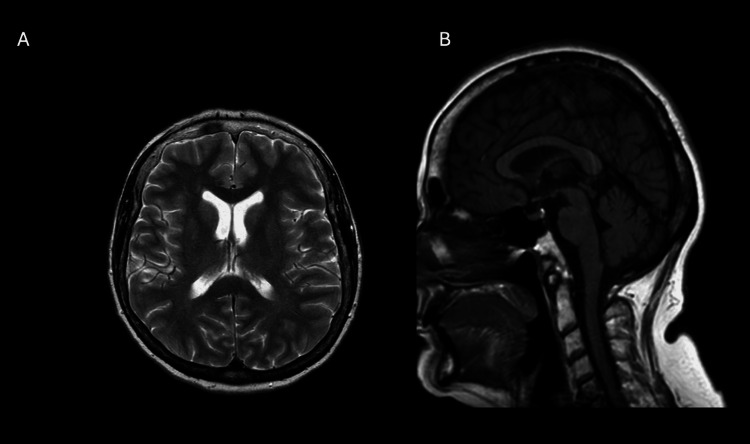
Repeat MRI head demonstrating no abnormalities (A) Axial T2-weighted view and (B) T1-weighted sagittal view.

**Figure 6:**
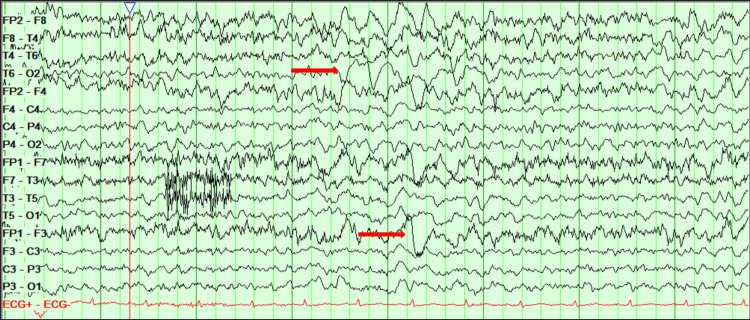
EEG displaying encephalopathic changes labeled by the red arrows EEG: Electroencephalogram.

A repeat CT brain demonstrated no acute intracranial abnormalities, and MRI of the head was inconclusive (Figures 4, 5). An electroencephalogram (EEG) demonstrated epileptiform discharges at the frontal region on both sides, along with encephalopathic changes (Figure 6). She was treated with an antiepileptic in the form of lamotrigine and aciclovir (antiviral) for suspected encephalitis.

A lumbar puncture was performed, which showed clear, colorless cerebrospinal fluid (CSF), normal CSF glucose and proteins with no organisms on Gram staining, and no oligoclonal bands. Additional antiviral screens from the CSF, including Herpes simplex virus (HSV) types 1 and 2 DNA, Varicella-zoster (VZ) DNA, enterovirus RNA, parechovirus RNA, and cytomegalovirus (CMV), were negative.

**Figure 7:**
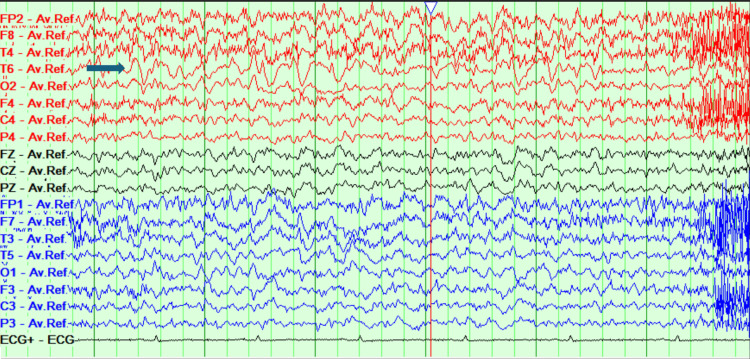
EEG displaying persistent epileptiform focus in the right posterior temporal region labeled by the blue arrow EEG: Electroencephalogram.

Her care was taken over by the neurology team, where she was treated for probable autoimmune encephalitis pending autoantibody screen from the CSF, with high-dose steroids (prednisolone 60 mg), and aciclovir (antiviral) was discontinued. She initially demonstrated improvement in her neurological symptoms, appearing less anxious and more engaged in conversation. As a result, her antipsychotic dosage (olanzapine) was gradually tapered over six days and subsequently discontinued. An MRI head was repeated, which demonstrated normal brain parenchyma. However, a repeat EEG displayed mixed-frequency and a slow and disorganized brain activity with a persistent and continuous epileptiform focus appearing in the right posterior temporal region, suggesting the development of focal non-convulsive status epilepticus (Figure 7).

**Figure 8:**
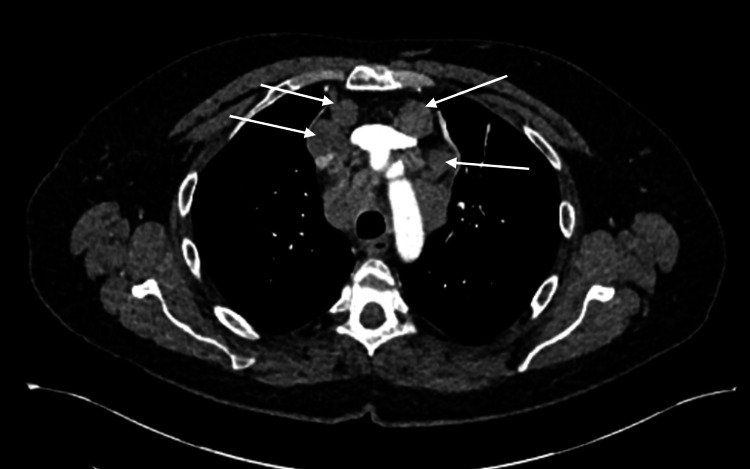
Extensive mediastinal adenopathy within multiple conglomerate mediastinal nodal enlargement labeled by the white arrows

The patient’s clinical symptoms began to deteriorate over a period of three to four days with increasing aggression, irritability, and unwillingness to engage with her care, and olanzapine (antipsychotic) was thus restarted. After a second opinion was sought, a CT thorax, abdomen, and pelvis (CT TAP) was performed, revealing extensive mediastinal adenopathy with no lung changes. Lymphoma was considered in the differential, and an endobronchial ultrasound (EBUS) was recommended for histological assessment (Figure 8). A repeat EEG demonstrated some improvement compared to her previous EEG, with no evidence of status epilepticus. She was treated with plasma exchange on alternate days, and Section 3 was rescinded due to clinical improvement in her behavior, where she appeared less irritable and anxious following the resumption of antipsychotics. Her steroids (prednisolone) were also weaned by 10 mg every two weeks.

She continued, however, to have seizures, and the dosage of lamotrigine was titrated up. A repeat MRI of the brain was normal, and updated autoantibody results from the CSF demonstrated that N-methyl-D-aspartate (NMDA) receptor, leucine-rich glioma-inactivated 1 (LGI1), contactin-associated protein 2 (CASPR2), AMPA1, AMPA2, and paraneoplastic antibody profile (Hu, Yo, Ri, Ma2 Ta, CV2, amphiphysin) were all negative.

She subsequently developed sepsis secondary to a central line infection, accompanied by worsening neurological symptoms in the form of increased aggression and hostility. As a result, plasma exchange was discontinued after completing three out of five planned sessions, and EBUS was delayed. She was then treated with intravenous immunoglobulins (IVIG) over a period of five days, leading to an improvement in her neurological symptoms.

The patient then began to complain of a two-week history of shortness of breath and concomitant fluctuating hoarseness of her voice. A flexible nasal endoscopy (FNE) and fiberoptic endoscopic evaluation of swallowing (FEES) were performed, which demonstrated left vocal cord immobility secondary to recurrent laryngeal nerve palsy caused by mediastinal lesions and severe bilateral edema (Table 2).

**Table 2:** FEES report showing abnormalities FEES: Fiberoptic endoscopic evaluation of swallowing.

Structures	
Nasal passage	Slightly tight, otherwise no abnormality detected (NAD).
Nasopharyngeal wall	Mild edema, watery salivary secretions present.
Velopharyngeal sphincter	Adequate conical closure on phonation and swallow.
Base of tongue	Reduced tongue base retraction, more on the right (R) side than the left (L), and thrush is present.
Oropharynx and posterior pharyngeal wall	Tented appearance. Secondary to respiratory dysfunction, worse on the right (R) side than the left (L). Slightly edematous appearance. Limited contraction on high-pitched “ee,” which improved with falsetto.
Epiglottis	Asymmetrical curve appearance with slight collapse on the right side as a result of pharyngo-epiglottic edema, R worse than L.
Valleculae space	NAD
Arytenoids	Severe bilateral edema with no movement of the left arytenoid, which appeared prolapsed. Unable to visualize the posterior third of the L vocal cord.
Aryepiglottic folds	Severe bilateral edema, no bands visible.
True vocal fold	White coating on the true cords, initially bilateral. Present at the anterior commissure and left lateral edge. Attempts were made to clear it; some material was mobilized; however, the white patch on the lateral edge of the left cord remained static. The left vocal cord showed palsy in a paramedian-to-midline position. The right vocal cord was mobile but limited by surrounding edema. Glottic closure was achieved on breath-hold. Edema of the right true cord was evident.
Ventricular folds	Severe bilateral edema
Pyriform sinuses	Severe edema, significantly reduced ability to hold residue
Cricopharyngeus (CP)	Edema above CP

**Figure 9:**
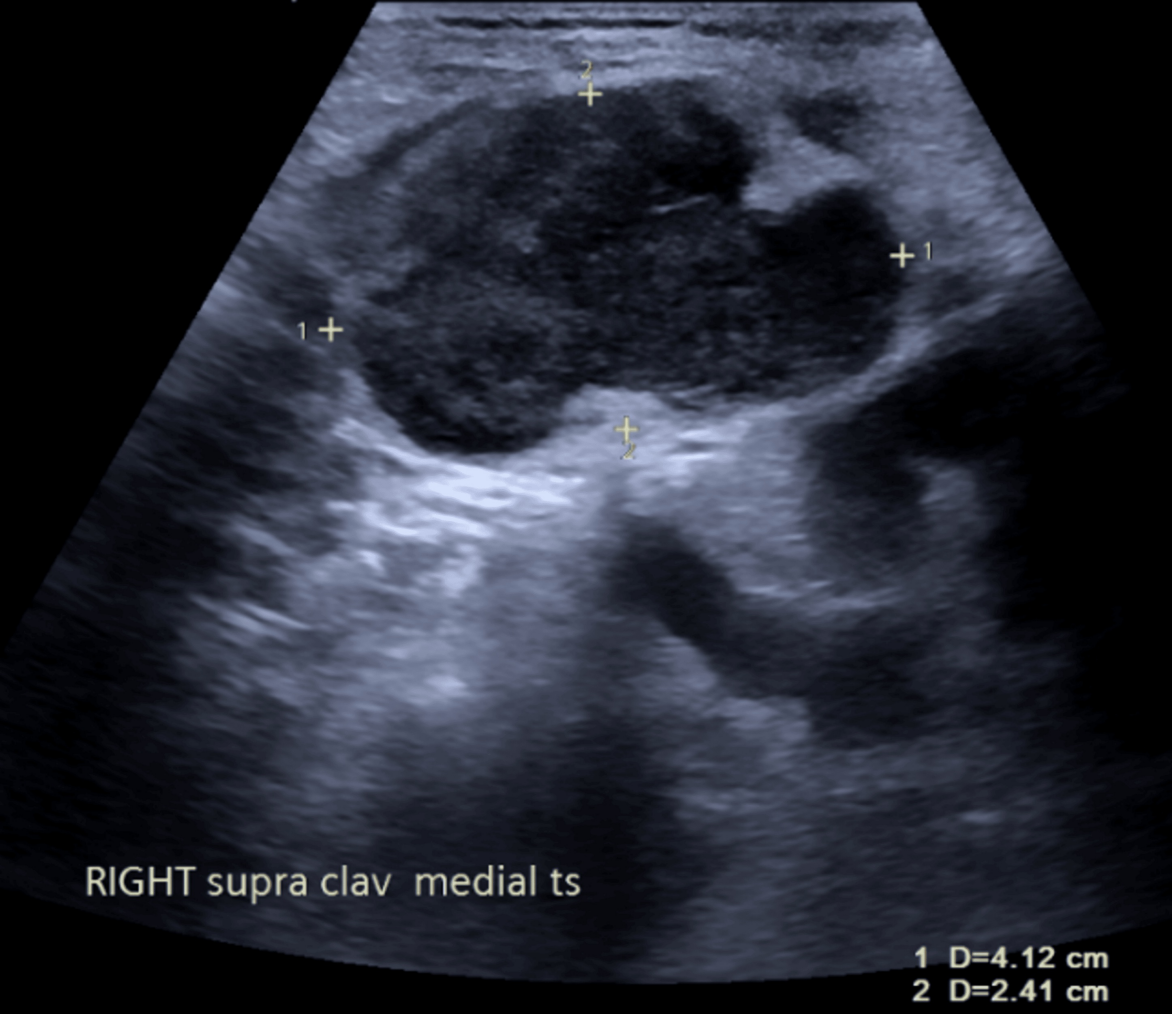
USS neck demonstrating lesion measuring 4.1 x 2.4 x 4.0 cm USS: Ultrasound scan.

Over the next few days, she developed right-sided neck swelling, facial redness, and prominent mediastinal veins. An ultrasound of the neck showed that the right subclavian vein (SCV) and right jugular vein were patent and compressible; however, superior to the medial part of the clavicle within the soft tissue, there was a mixed-echo lesion measuring 4.1 × 2.4 × 4.0 cm (Figure 9).

**Figure 10:**
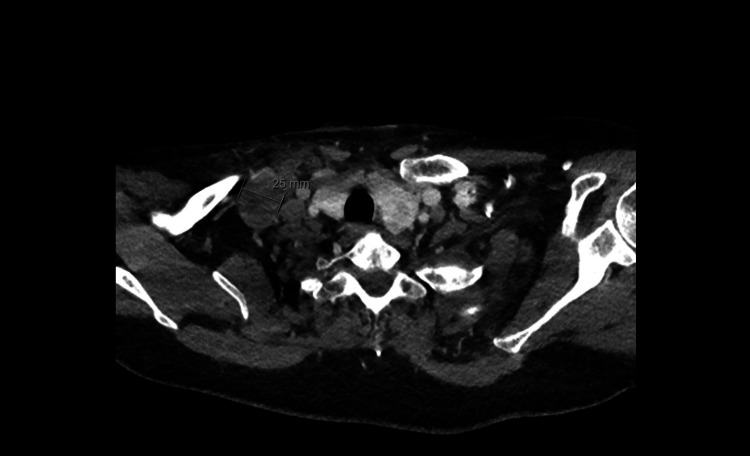
CT neck demonstrating large lymph nodal mass in the right lower cervical region measuring 2.5 cm

**Figure 11:**
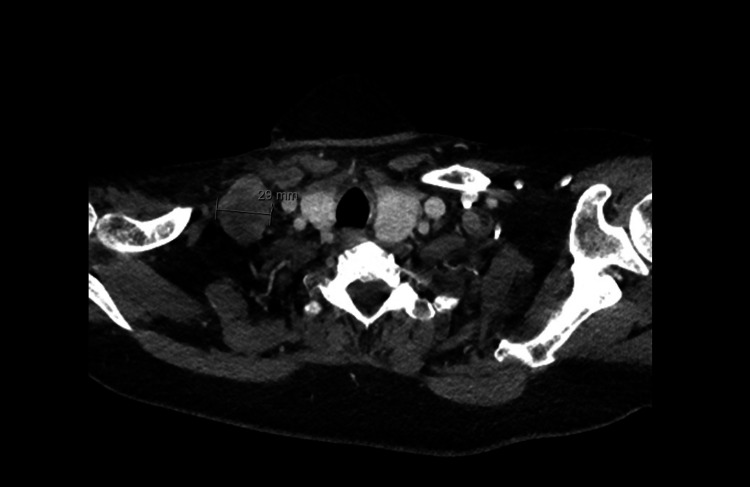
CT neck demonstrating large lymph nodal mass in the right lower cervical region measuring 2.9 cm

**Figure 12:**
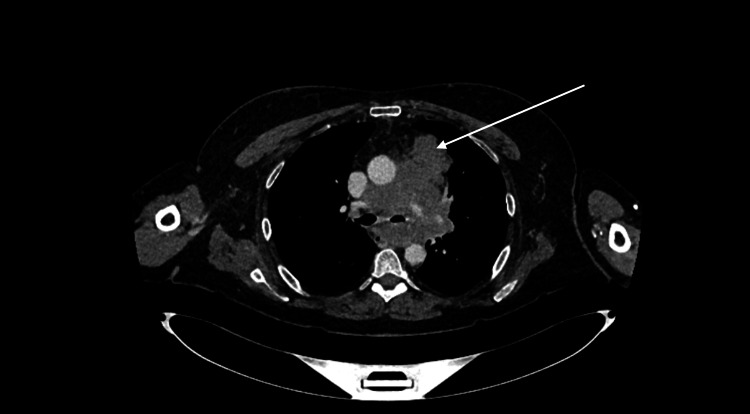
CT neck demonstrating progressive mediastinal lymphadenopathy labeled by the white arrow

A CT scan of the neck demonstrated multiple bilateral lower cervical lymph nodes (Figures 10, 11), with progressive confluent mediastinal lymphadenopathy compared with previous imaging (Figure 12).

EBUS was performed, and histology was reported as SCLC, staining positive for CK7, CAM5.2, and CD56, with strong nuclear positivity for TTF-1. The tumor cells were positive for synaptophysin and showed focal paranuclear dot-like positivity for chromogranin A. A CT scan of the abdomen and pelvis was performed for staging and revealed no lesions suspicious for metastasis and no abdominal or pelvic lymphadenopathy (cTxN3Mx).

**Figure 13:**
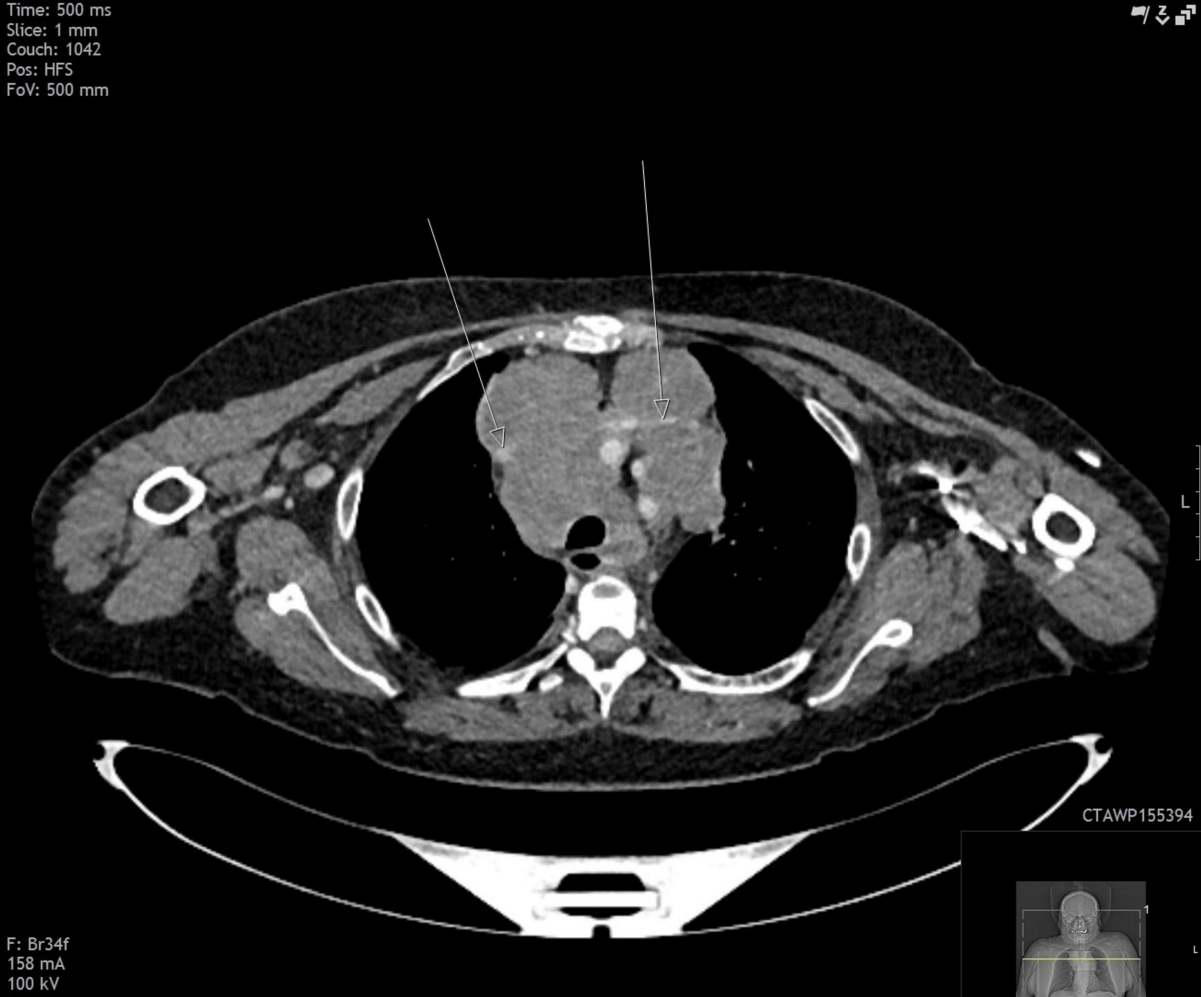
Axial CT demonstrating obstruction of bilateral brachiocephalic veins (labeled by the arrows)

The swelling in her right neck continued to increase in size, accompanied by new swelling on the left side of the neck, along with worsening shortness of breath. The previous CT neck was reviewed with an interventional radiologist, who noted that the brachiocephalic veins were bilaterally compressed due to the mediastinal mass. Although the superior vena cava (SVC) was not directly obstructed, there were signs of indirect SVC obstruction due to compression of these veins (Figure 13).

She was transferred to oncology and started on urgent chemotherapy with etoposide and carboplatin, administered in three-weekly cycles. Lamotrigine was discontinued, given no seizure activity; however, she remained on her psychotropic medications (olanzapine, fluoxetine, and clonazepam) due to their beneficial effects and history of previous treatment failure. The decision was made not to commence checkpoint inhibitor immunotherapy due to her underlying significant history of psoriasis, recent autoimmune encephalitis causing seizures and psychosis, and marginal performance status (ECOG2) limited by impending SVCO.

The patient's breathlessness, neck, and facial swelling improved significantly following chemotherapy, and her psychiatric symptoms resolved completely with no variation in behavior or mood. She was discharged from the hospital one week post-chemotherapy with a steroid weaning plan. Prednisolone was switched to dexamethasone 8 mg during her first cycle of chemotherapy and tapered by 2 mg every two days. She then resumed maintenance prednisolone at 20 mg thereafter, with a plan to reduce the dose by 10 mg every two weeks.

She received a further five cycles of chemotherapy as an outpatient alongside 19 days of thoracic and bilateral lower cervical radiation therapy, alongside cycle 5. Her latest follow-up (three months post-discharge) described her disease in near complete remission with no recurrence of psychiatric symptoms, and the plan is for prophylactic cranial irradiation to reduce the risk of brain metastasis following completion of her sixth cycle. Her psychotropic medications are due for review by the community psychiatric team, with consideration for weaning in light of the considerable improvement in her psychiatric symptoms.

## Discussion

PNS is a group of immune-mediated diseases secondary to underlying malignancy [10]. It is known that while anti-neuronal antibodies can be helpful in the diagnosis of PNS, 25%-32% of patients may not have them, making it difficult to suspect the condition [11].

This case highlights the unique presentation of progressive SCLC presenting with subacute neuropsychiatric symptoms, negative CSF reports, and brain imaging, but positive EEG changes. Despite ongoing treatment, sustained resolution of neuropsychiatric symptoms did not occur until after the initiation of chemotherapy, and as such, the diagnosis of paraneoplastic seronegative encephalitis was made.

The diagnosis of paraneoplastic encephalitis proves challenging due to its clinical manifestation resembling that of other mental or neurological conditions, including viral encephalitis, delirium, dementia, and other metabolic encephalopathies [8]. This patient had a varied clinical presentation during her admission. Her primary problems were confusion, manic psychosis, and seizures. Common organic causes of this presentation were taken into consideration, and she was treated for them. She was given antibiotics for a urinary tract infection, Pabrinex vitamins B and C for alcohol excess, and later treated as autoimmune encephalitis, following which the above symptoms briefly improved.

This patient also then developed impending SVCO​​​​​​, which is usually diagnosed clinically along with imaging studies. More than half of SVCO cases present due to an underlying malignancy, most commonly lung cancer. Out of these cases, half of them are due to non-small-cell lung cancer (NSCLC), and 25% are due to SCLC [12]. This prompted urgent chemotherapy, which then showed marked improvement in both the SVCO presentation and neuropsychiatric symptoms.

Notably, the diagnosis of small-cell lung carcinoma was not made until a CT TAP was performed, following which the mediastinal adenopathy was revealed, which then warranted an EBUS. The EBUS procedure was also unfortunately delayed due to sepsis secondary to a central line infection. The previous CT and MRI scans of the head were also unremarkable. Up to half of the patients with encephalitis of autoimmune origin would have a normal MRI, and in these cases, positron emission tomography (PET) may help identify signs suggestive of autoimmune encephalitis earlier than an MRI [10].

Although there were limitations that hindered the process of diagnosis, putting all of the above symptoms together and suspecting a paraneoplastic syndrome could have resulted in an earlier diagnosis of malignancy. However, in clinical practice, it is often difficult to balance the invasiveness, resource constraints, and logistical limitations of necessary testing with the goal of providing optimal patient care and timely detection of disease progression.

## Conclusions

Ultimately, this case highlights the importance of early recognition of differential diagnoses when treating a patient with a complex neurological presentation. PNS is difficult to diagnose in any patient. Nevertheless, this case illustrates that by investigating thoroughly, clinicians may detect and limit the progression of complications such as impending SVCO earlier. It is vital to include prompt treatment and enhanced diagnostic pathways to lower morbidity, thus improving patient outcomes. It is difficult to control all limiting factors, such as the patient becoming unwell prior to an invasive diagnostic procedure. However, resolving as many delays as possible is imperative to ensure a good standard of care.

## References

[1] Govindan R, Page N, Morgensztern D (2006). Changing epidemiology of small-cell lung cancer in the United States over the last 30 years: analysis of the surveillance, epidemiologic, and end results database. J Clin Oncol.

[2] Sculier JP, Evans WK, Feld R (1986). Superior vena caval obstruction syndrome in small cell lung cancer. Cancer.

[3] Kane RC, Cohen MH (1976). Superior vena caval obstruction due to small-cell anaplastic lung carcinoma. Response to chemotherapy. JAMA.

[4] Soomro Z, Youssef M, Yust-Katz S, Jalali A, Patel AJ, Mandel J (2020). Paraneoplastic syndromes in small cell lung cancer. J Thorac Dis.

[5] Alamowitch S, Graus F, Uchuya M, Reñé R, Bescansa E, Delattre JY (1997). Limbic encephalitis and small cell lung cancer. Clinical and immunological features. Brain.

[6] Fahim A, Butt M, McGivern DV (2010). A case of limbic encephalitis presenting as a paraneoplastic manifestation of limited stage small cell lung cancer: a case report. J Med Case Rep.

[7] Ryu JY, Lee SH, Lee EJ (2012). A case of paraneoplastic limbic encephalitis associated with small cell lung cancer. Tuberc Respir Dis (Seoul).

[8] Letargo J, Qu XM, Nguyen TK, Louie AV, Kuruvilla S, Kotrri E (2025). A neuropsychiatric prelude to unveiling small cell lung cancer with suspected paraneoplastic limbic encephalitis: a case report. Curr Oncol.

[9] Sun M, Chen X, Li H (2021). Clinical analysis of 48 cases of malignant superior vena cava syndrome. World J Surg Oncol.

[10] Vaišvilas M, Ciano-Petersen NL, Macarena Villagrán-García MD, Muñiz-Castrillo S, Vogrig A, Honnorat J (2023). Paraneoplastic encephalitis: clinically based approach on diagnosis and management. Postgrad Med J.

[11] Tsunoda Y, Kiwamoto T, Homma S (2017). Paraneoplastic limbic encephalitis with late-onset magnetic resonance imaging findings: a case report. Mol Clin Oncol.

[12] Rowell NP, Gleeson FV (2002). Steroids, radiotherapy, chemotherapy and stents for superior vena caval obstruction in carcinoma of the bronchus: a systematic review. Clin Oncol (R Coll Radiol).

